# The impact of structured decision making on absconding by forensic psychiatric patients: results from an A-B design study

**DOI:** 10.1186/s12888-015-0474-1

**Published:** 2015-05-03

**Authors:** Alexander I F Simpson, Stephanie R Penney, Stephanie Fernane, Treena Wilkie

**Affiliations:** Complex Mental Illness Program, Centre for Addiction and Mental Health, 1001 Queen Street West, Toronto, M6J 1H4 ON Canada; University of Toronto, Toronto, Canada

**Keywords:** Absconding, Risk, Forensic mental health, Policies and procedures, Intervention

## Abstract

**Background:**

Few studies have investigated absconding from forensic hospitals and there are no published studies of interventions aimed at reducing these incidents in forensic settings. We present a study of the impact of a new policy using structured professional judgment and an interdisciplinary team-based approach to granting privileges to forensic patients. We assess the impact of this policy on the rate and type of absconding from a metropolitan forensic facility.

**Methods:**

Following concern about the rate of absconding at our hospital, a new policy was implemented to guide the process of granting hospital grounds and community access privileges. Employing an A-B design, we investigated the rate, characteristics, and motivations of absconding events in the 18 months prior to, and 18 months following, implementation of this policy to assess its effectiveness.

**Results:**

Eighty-six patients were responsible for 188 incidents of absconding during the 42-month study window. The rate of absconding decreased progressively from 17.8% of all patients at risk prior to implementation of the new policy, to 13.8% during implementation, and further to 12.0% following implementation. There was a differential impact of the policy on absconding events, in that the greatest reduction was witnessed in absconsions occurring from unaccompanied passes; this was offset, to some extent, by an increase in absconding occurring from within hospital units or from staff accompanied outings. Seven of the absconding events included incidents of minor violence, and two included the commission of other illegal behaviors. The most common reported motive for absconding across the time periods studied was a sense of boredom or frustration. Discharge rate from hospital was 22.9% prior to the implementation of the policy to 22.7% after its introduction, indicating no change in the rate of patients’ eventual community reintegration.

**Conclusions:**

A structured and team-based approach to decision making regarding hospital grounds and community access privileges appeared to reduce the overall rate of absconding without slowing community reintegration of forensic patients.

**Electronic supplementary material:**

The online version of this article (doi:10.1186/s12888-015-0474-1) contains supplementary material, which is available to authorized users.

## Background

Absconding from forensic institutions presents significant clinical and reputational risks for those institutions, including a heightened perception of risk to public safety as well as a decreased sense of confidence in the efficacy of psychiatric services being provided [1-3]. Yet progressively increasing freedom of movement and community reintegration is a vital part of recovery for forensic service users, tasking the forensic mental health system with balancing the recovery-based and treatment needs of its clients with the larger obligation to protect the public from undue risk of harm [4,5].

Despite the significance and apprehension often associated with absconding events, we know little about how often these events occur. In their systematic review, Bowers and colleagues [6] reported the mean rate of absconding for general psychiatry (excluding forensic services) was 12.6%, calculated dividing the number of absconders by the number of inpatients at the beginning of the study plus those admitted over the course of the study (as per [7]), with a range of 2-44%. Studies from secure forensic hospitals in the U.K. report lower prevalence rates between 1-4% of all admissions [8-11]. Stewart and Bowers [12] reviewed 11 published studies of absconding from forensic facilities, finding a median rate of 0.76 absconding events per month per 100 beds (range 0.04-1.06). Of note, some of these studies are dated and from high security hospitals, not analogous to most modern forensic facilities of medium or minimum security associated with larger hospitals or general mental health units. Recently, we [13] published a case–control study of absconding among forensic patients within a large urban psychiatric hospital, finding an overall rate of 14.4% over a 24-month period.

Also not well described in the literature are the motivations driving absconding behavior, particularly among forensic patients. Bowers and colleagues [6] summarized the general mental health literature relating to patient-reported reasons for absconding. Themes that emerged included a propensity towards impulsive or noncompliant behavior, a sense of treatment failure or disagreement about the need for hospitalization, a widespread sense of boredom and frustration, as well as family problems or a lack of family involvement [6,14]. For others, absconding was reflective of goal-directed behavior, either to complete a task (e.g., related to household responsibilities) or to obtain substances. A recent study of psychiatric patients who absconded in Iran found similar motivational themes, including feelings of boredom as well as missing family members [15]. Few studies have found evidence of symptom-driven motivation when patients are asked directly about their reasons for absconding (e.g., [14]); however, fear and safety concerns were noted to play a significant role in patients’ decisions to abscond, as were failures in the therapeutic relationship with staff [16]. In forensic samples, patients who absconded endorsed a desire to be at liberty [9], and expressed feelings of frustration related to long periods of detention and a sense that they were not making progress and being allowed greater freedoms [13]. In this latter study, we further found evidence of a group of patients whose absconding appeared primarily motivated by symptoms of illness or behavioral disorganization, as well as a group who absconded mainly or exclusively to complete a specific task, but did not have the required privileges to do so at the time.

### Assessing risk and designing interventions to reduce absconding in forensic settings

Existing literature suggests that certain clinical and risk management issues involved in the care of forensic patients are distinct from general mental health patients [5,13]. All forensic patients are subject to compulsory detention, and many for prolonged periods of time. In addition to problems of serious mental disorder, they commonly also have difficulties of treatment engagement, substance misuse and antisocial behavior. They are overwhelmingly male, of minority ethnicity, and often young. Owing in part to differences in risk and hospital length of stay that distinguish forensic from general mental health patients, the corresponding characteristics of and motivations involved in their absconding behavior will likely differ as well. Interventions designed to reduce incidents of absconding must therefore understand the multiple and often unique determinants for this behavior among forensic populations, and the need for individualized risk formulation and management.

Unfortunately, despite significant advancements in the field of violence risk assessment, there currently exists just one unpublished guideline designed to assess risk for absconding (the leave/absconding risk assessment (LARA; [17]). Hilterman, Philipse, and Graaf [18] published a tool to assess the risk of violence upon discharge or unauthorized leave in the community but this tool does not address the risk of absconding per se. That said, the characteristics of patients who abscond from secure settings overlap with many empirically-based risk factors for violence (e.g., male gender, active or recent psychiatric symptoms and treatment non-compliance, employment and substance use problems, impulsivity; [19,20]), such that a logical approach could be to utilize knowledge from the violence risk assessment field to inform decisions about risk for absconding, and in turn, decisions regarding leave for forensic patients. The use of structured professional judgment tools such as the Historical, Clinical, and Risk Management-20 (HCR-20; [21]), while designed to inform judgments of risk for future violence, may be of use in assessing individuals who are at risk for absconding. In fact, the HCR-20 has been shown to be useful in assessing risk for a wide array of adverse outcomes (e.g., non-violent reoffending, hospital readmission, progress in treatment, non-compliance with jail diversion programs; [22-25]), suggesting that it is not limited to violence. Other well-studied clinical assessment measures that have shown utility in the prediction of violence and other adverse outcomes may similarly be of use (e.g., the Psychopathy Checklist, Revised; PCL-R [26]).

Existing approaches within forensic services to assessing absconding risk and making decisions about progression to greater degrees of liberty are diverse [27,28], likely reflecting the absence of standardized tools for this purpose. Within this context, we also know little about how clinical teams make decisions regarding the granting of different levels of privilege. Lyall and Bartlett [29] investigated the decision-making processes of two forensic mental health teams over a 15-month period. They found that leave decisions were not made in a structured way, and that the risk of absconding was often not explicitly discussed. Stubner et al. [30] surveyed clinicians in 12 German forensic hospitals and found that most institutions did not employ any type of structured checklist or set of criteria to guide decisions regarding when patients could receive increased freedoms. However, variables related to a patient’s recent functioning (e.g., current symptoms of mental disorder or substance use, treatment alliance) were consistently of more influence when making leave decisions as compared to historical or static risk factors. These findings add support to the likely utility of a structured risk assessment approach, such as that embodied by the HCR-20, both for ensuring teams attend to all relevant factors in their assessment of risk, as well as increasing the transparency and consistency of leave decisions. Furthermore, a focus on recent and modifiable risk factors may have most relevance for assessing a patient’s absconding risk, given that these variables are shown to perform better when predicting short-term adverse outcomes (e.g., inpatient aggression; [31,32]) as compared to historical or static ones that have differentiated absconders from non-absconders in prior investigations.

Given the relative paucity of prospective research on risk factors for absconding, it is perhaps unsurprising that there are few well designed studies of interventions to reduce absconding [6], and none in the forensic literature. Bowers and colleagues [33,34] designed and evaluated an intervention which encompassed six measures to deter absconding, including the use of a sign in/out book to increase clarity of rules surrounding leave, supportive breaking of bad news to patients, debriefing following any aggressive incident on the ward, multidisciplinary review after two absconding incidents by the same patient, and identifying and targeting nursing time to those at high risk of absconding. Using an A-B design of short duration (three months before and after the intervention) they found a 25% reduction in absconding rates in acute and general mental health wards. However, there was noted variation among the wards studied, as well as an unexpected increase in violence during the intervention (upon closer examination this increase was found to be caused by one particular patient who was difficult to manage).

The absence of studies evaluating policies or interventions designed to reduce absconding in forensic settings also reflects the difficulty in designing and implementing such investigations. Security issues in relation to forensic patients mean that randomization (e.g., granting different levels of community access to different patients on a random basis) is not ethically appropriate. Similarly, delaying interventions in a staged process would not be legally appropriate, and blindedness is not feasible as staff and patients must be informed about the conditions of their leave as it is available to them. Thus, implementation studies of an A-B design are likely to be the best studies that can be performed within forensic settings. We recently had the opportunity to conduct such a design given the implementation of a new policy designed to reduce incidents of absconding occurring in our forensic program. The policy involved the use of structured professional judgment based on the HCR-20 and an interdisciplinary team-based approach to granting privileges for forensic patients.

### The current study

This study investigates the impact of a new policy designed to reduce incidents of absconding in a forensic setting. Employing an A-B design, we describe the rate of absconding in the 18 months prior to, and 18 months following, implementation of this policy to assess its effectiveness. We further investigate key clinical events transpiring in the month prior to an absconding event, as well as during the event itself. Relevant sociodemographic, legal and clinical characteristics of patients who absconded during the study were coded, as were the motivational influences that appeared to be driving the absconding behavior. As such, we were able to investigate the impact of the new policy on overall rates of absconding, as well as whether it had a differential impact on certain types of absconding events or types of patients who abscond. We further investigate differences between patients who absconded during the study window to those who did not, building on findings presented in an earlier study [13].

We expected that the rate of absconding would show a significant decline in the months following institution of the new policy, as compared to the time period preceding and during its implementation. Further, we expected there to be a high rate of boredom and frustration cited as key motivational influences underlying the absconding behavior witnessed. Given the lack of prior study in this area, we did not have direct hypotheses regarding the differential impact of the policy on specific types of absconding events or patients who abscond. We expected to see similar differences between absconding patients and controls to those witnessed in our earlier study (e.g., longer lengths of stay under the forensic mental health system, as well as higher risk scores on the HCR-20 for the absconding group), but with stronger effect sizes given the larger sample size available in this study.

## Methods

### Development of a new policy

Concern arose in our facility in 2012 regarding the frequency of absconding incidents. Although there had been no serious safety incidents during any prior absconsions, the risks associated with a high rate of absconsion was perceived as clinically and organizationally hazardous. A project group was established to perform a comprehensive literature review and survey of policies and procedures employed at our facility as well as other hospitals, and was led by one of us (AS; clinical director of the forensic program). Two of the study authors (AS and TW) are psychiatrists in the program and are routinely involved in making risk decisions as they pertain to the granting of leave privileges for patients.

The literature review and survey of policies revealed gaps in our practice. First, it was noted that many units were not employing a team-based decision making process, but rather decisions were being made by the attending psychiatrist and ward manager outside of team meetings. Second, referral of key decisions regarding privileges to hospital administration was encouraged but did not occur on a consistent basis. Third, practices regarding debriefs and review following absconding incidents were inconsistent across teams. As there were no published instruments in the scientific literature, a tool was designed (Leave Application Form; Additional file: 1) to remedy these identified gaps in practice and, specifically, to assist clinical teams arrive at decisions regarding leave in a manner informed by the risk literature more broadly. The form guides clinicians through three steps of making an application for a new level of privilege category:

1. Integrate risk indicators from the HCR-20 related to past rule and supervision violations, substance use, and current level of insight and clinical stability;
2. Define the specific nature and purpose of the leave being sought, including how the leave relates to, or will facilitate, existing individualized rehabilitation goals;
3. Define the risks and benefits of the leave, including specific victim related issues, the patient’s attitude towards the leave, and the risks of not granting it.

The Leave Application Form was to be completed during the multi-disciplinary team meetings held each week on the unit, and to have the input of all team members. Guided by examples of good clinical practice from other centres, a second tier of review for the forms was established. A committee of senior clinical and managerial staff was formed to consider, approve, or decline these requests. Policy was clarified regarding the levels of privilege that required approval from the Person in Charge (a senior clinician with statutory authority to create and approve rehabilitation plans for patients), and what to do if privileges were suspended or a patient absconded. Privileges were approved by category (e.g., unescorted hospital grounds, escorted community), and clinical teams could then grant individual instances of leave within the approved category, and increase their length and frequency, according to the patient’s needs and progress.

All leaves were initially restricted in March 2012, following concerns regarding a high frequency of absconding. Teams were instructed to be more vigilant regarding the granting of privileges while the new policy and form were developed. Different versions of the Leave Application Form were trialed, and feedback was gathered regarding areas needing improved clarity. The senior review process was established while teams were adjusting their practices to integrate the new form into their team meetings. This process was complete across all of the forensic units at our institution by August 2012.

### Setting

The study was conducted at a large urban psychiatric hospital in Toronto, Canada, closely integrated with the surrounding downtown community. The forensic program within this hospital is comprised of 180 inpatient beds divided between four medium (82 beds) and four minimum (98 beds) secure units and serves approximately 250 community forensic outpatients (i.e., patients who continue to be under the auspices of the Ontario Review Board [ORB] but have been granted a conditional discharge or community living privileges). Almost all patients have been found Not Criminally Responsible on account of Mental Disorder (NCRMD) and all are under the auspices of the ORB. At the time of the study, the most common index offense in our patient population was assault (58%), followed by uttering threats (20%) and weapons charges (15%). Twelve percent of patients had been charged with a sexual offense, while 13% were charged with murder (7%) or attempted murder (6%). Eight percent had only a non-violent (e.g., property) offense for which they were found NCRMD.

The ORB is responsible for reviewing the status of every person under its jurisdiction on an annual basis, and making decisions regarding the least restrictive placement of the individual for the forthcoming year (i.e., continued detention, conditional or absolute discharge from hospital) with regards to public safety. Within the limits set by these annual decisions, patients under the ORB have access to a graded range of passes from secure units. The hospital, or statutorily the Person in Charge, grants the progression of these passes, initially allowing access to hospital grounds only (from escorted on to accompanied and then unaccompanied passes) and progressing into the community (also escorted, accompanied and unaccompanied passes, up to and including overnight passes). It is the role of the Person in Charge to grant privileges according the patient’s clinical progress and any risk issues that may emerge, within the limits set by the patient’s ORB disposition.

### Design

We studied the rate and characteristics of absconding events during the 18 months preceding the implementation of the new policy in our program, lasting from September 1, 2010 to February 29, 2012 (the ‘A’, or pre-period). We then compared the rate and characteristics of absconding events occurring from September 1, 2012 to February 28, 2014, inclusive (the ‘B’ or post-period), as well as all incidents occurring in between these two phases. All absconding events were identified from three separate sources (daily progress notes, incident reports, and required email communications when a patient absconds). Consistent with prior research, we defined absconding as any unauthorized absence from the hospital. This included breaching the security of an inpatient unit, accessing hospital grounds or the community without permission, or being absent for longer than permitted. The study was approved by the Centre for Addiction and Mental Health ethics review board prior to the commencement of data collection. Because the data collected was archival in nature, written consent from patients was not required.

### Measures

We developed a coding form to gather all relevant sociodemographic, legal and clinical information for every patient with one or more incidents of absconding during the study period. Data pertaining to the month prior to the event (e.g., medication change or non-compliance, change in mental status, substance use, voiced ideation/intent to abscond), events transpiring during the unauthorized absence (e.g., involvement in or experience of violence, substance use), as well as characteristics of the absconsion itself (e.g., method of leave, duration, location traveled to, form of return to hospital) were recorded. All data were collected by one study author (SF) from the patient’s health record, including assessment and treatment reports, legal documents, as well as daily progress notes completed by nursing staff and other members of the clinical team. Information pertaining to patient motivation was collected from daily progress notes. These notes summarized the interaction that took place with the patient upon their return to the unit, including patients’ responses to being asked directly about why they absconded.

The HCR-20 [21] was used to compare risk levels of patients who absconded across the three time periods described above (i.e., A, B, interim). The HCR-20 is a 20-item violence risk assessment scheme for use with adults who have a history of violence as well as mental illness and/or personality disorder. Items appearing on the HCR-20 may be grouped thematically into historical/static risk factors, current clinical concerns, and future-oriented/risk management variables, and are coded on a 0 (*not present*), 1 (*possibly or partially present*), and 2 (*definitely present*) point scale. The Psychopathy Checklist, Revised (PCL-R; [26]) was used for similar purposes. It is a 20-item symptom construct rating scale designed to measure the interpersonal, affective, and behavioral characteristics of psychopathic personality disorder in adults. The items appearing on the PCL-R are scored on a 0, 1, 2 scale reflecting trait presence and severity.

### Data analysis

We calculated the rate of absconding from all of our forensic units for the pre- and post-policy implementation phases, as well as the six months in between, by dividing the number of patients who absconded during each time period by all current inpatients plus new admissions (as per [6,7,34]). We supplemented this with an event-based index of absconding, calculated as the number of absconding incidents divided by the number of bed days over which they were counted (beds x days), and then multiplying by 100 to produce the absconding rate per 100 bed days (as per [34]). The effect of the policy on rates of absconding across each of the three time periods was evaluated with a *z*-ratio for proportions (two-tailed). Statistical tests of difference (ANOVA [Kruskal-Wallis for variables with non-normal distributions], *χ*^2^) were used to compare the characteristics of absconding events, as well as the sociodemographic, legal and clinical profiles of absconding patients, before, during, and after the policy implementation.

Qualitative thematic analysis of patient motives for absconding was undertaken to investigate whether there was a differential impact of the new policy on motivational subtypes of absconding incidents. To do this, three study authors (SP, SF, and TW) independently read all available clinical information surrounding a client’s absconsion, including documentation of the patient’s self-reported motives. We then each rated what we judged to be the primary motivation(s) underlying the behavior; that is, the one or two variables that appeared to be functionally or causally related to their absconding. We subsequently met to discuss our ratings for each case. At this stage, each incident of absconding was assigned into one of four distinct and non-overlapping profiles of absconding behavior. We found that the existing motivational typologies created for our first study (i.e., goal-directed, frustration/boredom, symptomatic/disorganized, accidental/no intent; [13]) fit the current data well, and that no new categories needed to be created. Of note, patients with multiple absconding events could be classified into more than one group if the events were characterized by different motivational influences.

## Results

### Rate of absconding

Eighty-six patients were responsible for 188 incidents of absconding during the 42-month study window. Forty-six patients had single incidents, while 40 patients absconded on two or more occasions. Across the eight inpatient units studied, a higher number of absconding events resulted from patients residing on minimum secure units (78% of all events) as compared to patients on medium secure units (22%). This is to be expected, given that the level of hospital grounds and community access is significantly greater for patients residing on the former. The rate of absconding was calculated for each of three non-overlapping time periods, and indices of patient-based and event-based absconding are presented in Table 1. The number of patients absconding decreased by 33% (*p* < .05), while the number of incidents as measured by bed days decreased by 40% (*p* < .01). Discharge rate from hospital was 22.9% prior to the implementation of the policy to 22.7% after its introduction, indicating no change in the rate of patients’ eventual community reintegration.

**Table 1:** **Rate of absconding**

	***A*** **Sep 1 2010 – Feb 29 2012**	***Interim*** **Mar 1 2012– Aug 31 2012**	***B*** **Sep 1 2012 – Feb 28 2014**	**Diff A/B**	**Diff A vs. Interim**	**Diff B vs. Interim**
# patients absconding/# patients at-risk	17.8%	13.8%	12.0%	*z* = 2.08, *p* < .05	*z* = 1.21, *p* > .05	*z* = −0.60, *p* > .05
# absconding incidents/# bed days	0.10	0.14	0.06	*z* = 2.97, *p* < .01	*z* = −1.58, *p* > .05	*z* = 3.97, *p* < .01

Descriptive data pertaining to clinically relevant events occurring in the month prior to an incident of absconding are presented in Table 2, while characteristics of the absconding events are presented in Table 3. These data are again presented for each of the three time periods under investigation and are statistically compared to one another. Results show that there were recent changes in medication preceding over one-third of absconding events occurring prior to the implementation of the new policy, and this declined significantly during and following implementation. A significant reduction was also seen in the number of absconsions characterized by recent noncompliance with medication, noncompliance with privilege levels and passes, as well as preceding changes in privilege level. However, expressed ideation to abscond continued to precede over one-quarter of all events throughout the study window. There was a significant increase in the number of absconsions preceded by recent patient involvement in violent or threatening behaviors following the policy implementation. However, it is important to note that this increase was driven primarily by acts of nonverbal aggression/threatening, rather than serious physical forms of violence.

**Table 2:** **Events occurring in the month prior to absconsion - % of all incidents (** ***N*** **= 188)**

	***A*** **Sept 1 2010 – Feb 29 2012 (** ***n*** **= 89)**	***Interim*** **Mar 1 2012 – Aug 31 2012 (** ***n*** **= 40)**	***B*** **Sept 1 2012 – Feb 28 2014 (** ***n*** **= 59)**	***χ*** ^***2***^
Medication change	38.2	10.0	22.0	18.74**
Noncompliance with medication	23.6	5.0	17.0	6.62*
Change in symptoms/mental status noted	32.6	17.5	25.4	3.30
Stressful/adverse event noted	32.6	17.5	27.1	3.15
Suicidal ideation expressed	3.4	2.5	8.5	--
Absconding ideation expressed	28.1	22.5	27.1	0.45
Attempted absconding	7.9	0.0	3.4	--
Noncompliance with privileges/passes	50.6	27.5	25.4	11.82**
Change in privilege level	93.3	72.5	57.6	26.74**
Engagement in violence (incl. threats)	18.0	42.5	37.4	10.70**
Engagement in substance use	14.6	15.0	5.1	3.65

*p < .05. **p < .01. The Freeman-Halton extension of the Fisher exact probability test (two-tailed) was performed alongside the *χ* ^*2*^ computation.

--*χ* ^*2*^ not calculated due to > 20% of cells having sample sizes < 5.

**Table 3:** **Characteristics of absconding incidents (** ***N*** **= 188)**

	***A*** **Sept 1 2010 – Feb 29 2012 (** ***n*** **= 89)**	***Interim*** **Mar 1 2012 – Aug 31 2012 (** ***n*** **= 40)**	***B*** **Sept 1 2012 – Feb 28 2014 (** ***n*** **= 59)**	
	**Mdn/M (SD)**	**Mdn/M (SD)**	**Mdn/M (SD)**	***KW***_***H***_
Duration (hours)	8.5/41.1 (95.5)	6.5/53.7 (174.2)	4.3/51.1 (144.7)	5.33
Method		**%**		***χ*** ^***2***^
Off locked unit	12.4	5.0	23.7	7.35*
From staff accompanied outing	12.4	10.0	28.8	8.57*
From unaccompanied hospital pass	41.6	65.0	33.9	9.81**
From unaccompanied community pass	30.3	15.0	13.6	7.24*
Location during leave		**%**		***χ*** ^***2***^
Within city limits, outdoors or public place	57.3	45.0	69.5	5.99*
Own home	11.2	0.0	3.4	--
Friends/family home	21.4	27.5	22.0	0.68
Shelter	13.5	12.5	5.1	2.82
Hospital grounds	9.0	27.5	20.3	7.79*
Substance use (yes)	31.5	35.0	27.1	0.72
Reoffense (yes)	2.3	0.0	0.0	--
Violence – perpetrator	2.3	2.5	6.8	--
Violence – victim	1.1	0.0	0.0	--
Form of return		**%**		***χ*** ^***2***^
Self	55.1	65.0	47.5	2.97
Police	33.7	25.0	28.8	1.08
Hospital staff	4.5	7.5	17.0	6.84*
Family member	3.4	2.5	3.4	--

*Note*. Outliers were removed in calculating the Duration variable: three incidents that lasted 117, 125, and 130 days, respectively. *KW*_*H*_ = Kruskal-Wallis H test.

*p < .05.**p ≤ .01. The Freeman-Halton extension of the Fisher exact probability test (two-tailed) was performed alongside the *χ* ^*2*^ computation.

--*χ* ^*2*^ not calculated due to > 20% of cells having sample sizes < 5.

Across the entire study window, the mean duration of absence was approximately 2 days (47 hours, Mdn = 7 hours), with a range of 10 minutes to 41 days (this excludes 3 patients who were absent for approximately 4 months each). Sixty-one percent of unauthorized absences were under 12 hours, while 72% were under 24 hours. There was little variation in the mean duration of absconding events across the three time periods studied, although the median value following the policy implementation decreased. Regarding the method of leave, results show a significant decrease in the number of absconding events occurring from unaccompanied hospital grounds and community passes following the policy implementation. In contrast, we observed a significant increase in the number of events arising from staff accompanied outings (whether on hospital grounds or in the community), as well as an increase in absconsions from within inpatient units. With the exception of one individual, all patients who absconded were returned to hospital.

Two criminal offenses occurred by two separate patients while absent. The first, a female patient, visited the home of the victim she had previously stalked on the basis of an erotomanic delusion. She had done this several times since coming to hospital. The second incident involved a male client who absconded during an outing to his immigration hearing, and was found by police later that evening in a restaurant where he was threatening other patrons. In seven cases, patients had engaged in some form of violence (including the incident described above). These incidents most commonly involved behaving aggressively (e.g., kicking, spitting) towards hospital staff or police who were trying to return the patient to hospital. In one incident, a patient knocked over a nurse and a housekeeper while fleeing the hospital; in another, a patient visited the home of his parents and was reported to have yelled and threatened them (this patient’s father was also the victim of his index offense). Substance use during absconding incidents occurred in approximately one-third of cases across the study window.

### Characteristics of patients who abscond

The sociodemographic, clinical and legal characteristics of absconders across the three periods are presented in Table 4. There were no significant differences across any of these variables, suggesting that the profile of absconders did not differ appreciably prior to or following implementation of the new policy. When removing those patients with multiple incidents of absconding who populated more than one time category, results were unchanged with the exception that patients absconding only within the interim time period were significantly older as compared to those patients absconding either before or after the policy implementation (*F* [2, 83] = 4.41, *p* < .05). Compared to patients with just a single incident of absconding, patients who absconded on multiple occasions were, on average, 6.5 years younger (*F* [1, 84] = 6.89, *p* = .01).

**Table 4:** **Demographic, clinical and legal characteristics of absconders (** ***N*** **= 125)**

	***A*** **Sept 1 2010 – Feb 29 2012 (** ***n*** **= 58)**		***Interim*** **Mar 1 2012 – Aug 31 2012 (** ***n*** **= 28)**		***B*** **Sept 1 2012 – Feb 28 2014 (** ***n*** **= 39)**		
	**M**	**SD**	**M**	**SD**	**M**	**SD**	***F*** **/** ***KW***_***H***_
Age	40.52	11.35	44.14	14.23	38.03	10.09	2.32
Days under ORB	2246.19	2107.07	2286.89	1313.41	2372.38	1513.56	1.74
PCL-R total score	18.76	6.21	18.59	5.67	19.46	5.25	0.17
HCR-20 total score	26.13	4.65	26.23	4.68	27.50	4.77	1.03
	**N**	**%**	**N**	**%**	**N**	**%**	***χ*** ^***2***^
Sex (male)	46	79.3	22	78.6	28	71.8	.80
Ethnicity							
Caucasian	29	52.7	14	53.8	16	50.0	3.24
Afro-Caribbean	17	30.9	10	38.5	14	43.8	
Asian	9	16.4	2	7.7	2	6.2	
History of absconding (yes)	33	58.9	20	71.4	28	71.8	2.19
Prior absconding attempts (yes)	18	35.3	7	25.0	13	33.3	0.91
Diagnosis							
Primary psychotic disorder	14	24.1	7	25.0	5	12.8	3.02
Comorbid psychosis + substance abuse	36	62.1	16	57.1	25	64.1	
Personality disorder indicated (yes)	24	41.4	10	35.7	16	41.0	0.28
Index offense							
Violent	38	65.5	20	71.4	27	69.2	0.88
Non-violent	11	19.0	4	14.3	5	12.8	
Sexual	9	15.5	4	14.3	7	17.9	

*Note*. ORB = Ontario Review Board; PCL-R = Psychopathy Checklist, Revised; HCR-20 = Historical, Clinical, Risk Management-20. Patients with more than one incident of absconding can fall into more than one time category.

We next investigated the frequency of absconding events characterized by different motivational influences. As shown in Table 5, the different motivational subtypes did not show significant fluctuation across time (the symptomatic/disorganized group showed a decrease at the trend level, *p* < .10). This suggests that the impact of the new policy was relatively equivalent across motivational influences. As mentioned above, we found that the existing motivational typologies created for our first study fit the current data well, and that no new categories needed to be created. The only novel element we identified was a notably high level of expressed negative affect (most often anger), corresponding to 42% of incidents within the frustration/boredom group.

**Table 5:** A**bsconding events based on primary motivation (** ***N*** **= 188)**

	***A*** **Sept 1 2010 – Feb 29 2012 (** ***n*** **= 89)**		***Interim*** **Mar 1 2012 – Aug 31 2012 (** ***n*** **= 40)**		***B*** **Sept 1 2012 – Feb 28 2014 (** ***n*** **= 59)**		***χ*** ^***2***^
	**N**	**%**	**N**	**%**	**N**	**%**	
Goal-directed	14	15.7	10	25.0	14	23.7	2.13
Frustration/Boredom	47	52.8	21	52.5	36	61.0	1.13
Symptomatic/Disorganized	25	28.1	7	17.5	8	13.6	4.91^†^
Accidental/No intent	3	3.4	2	5.0	1	1.7	--

^†^ *p* < .10.

--*χ* ^*2*^ not calculated due to > 20% of cells having sample sizes < 5.

Lastly, building on our earlier analyses comparing absconders to controls (matched on age, sex, and security level within the hospital), we re-ran these analyses using the current expanded sample (Table 6). Results were similar to our prior findings. In particular, patients with one or more incidents of absconding were characterized by significantly longer lengths of stay under the forensic mental health system, as well as higher risk scores on the HCR-20. Effects that were at the trend level (p < .10) previously reached significance in this larger sample (i.e., higher incidence of absconding attempts, more likely to have a comorbid substance use disorder as well as problematic personality traits or disorder).

**Table 6:** **Demographic, clinical and legal characteristics of absconders and controls**

	**Absconders (** ***N*** **= 91)**		**Controls (** ***N*** **= 88)**		
	**M**	**SD**	**M**	**SD**	***F*** **/** ***KW***_***H***_
Age^†^	40.76	12.20	39.86	11.02	0.27
Days under ORB	2199.33	1977.01	1325.16	1022.31	12.37**
PCL-R total score	18.40	6.20	16.13	6.79	3.80*
HCR-20 total score	26.00	5.09	21.90	6.07	22.10**
	**N**	**%**	**N**	**%**	***χ*** ^***2***^
Sex (male)^†^	69	83.0	73	75.8	1.39
Ethnicity					
Caucasian	44	53.7	25	32.9	7.87*
Afro-Caribbean	29	35.4	34	44.7	
Asian	9	11.0	17	22.4	
History of absconding (yes)^†^	51	57.3	0	0.0	69.55**
Prior absconding attempts (yes)	25	30.1	5	5.8	17.09**
Diagnosis					
Primary psychotic disorder	22	24.2	35	39.8	5.05*
Comorbid psychosis + substance abuse	55	60.4	43	48.9	
Personality disorder indicated (yes)	38	41.8	25	28.4	3.50*
Index offense					
Violent	65	71.4	58	65.9	0.77
Non-violent	16	17.6	17	19.3	
Sexual	10	11.0	13	14.8	

*Note*. ORB = Ontario Review Board; PCL-R = Psychopathy Checklist, Revised; HCR-20 = Historical, Clinical, Risk Management-20. The absconding group contains all non-overlapping clients who absconded between January 1, 2010 and February 28, 2014.

^†^Denotes a variable that was matched across absconding and control groups (age, sex), or held to = 0 in the control group (history of absconding).

*p < .05.**p ≤ .01.

## Discussion

Absconding from secure settings remains a significant clinical, institutional and public safety issue, yet there continue to be relatively little data that speak to the prevalence of these behaviors and the motivational influences that may be driving them. Also lacking are prospective investigations into the risk factors that are associated with a higher likelihood of absconding, as well as corresponding risk management and intervention strategies that could be employed to reduce rates of absconding from within forensic settings. The rate of absconding found in the current study was comparable to other general mental health settings [6,12,35], and is reflective of a rehabilitating forensic sample located within a large, urban, community-integrated hospital. Consistent with our earlier results, we continued to find a low rate of public harm, violence, and offending committed by patients who abscond.

Despite the absence of serious violence occurring during patient leaves, operating with a relatively elevated rate of absconding in a secure setting is both clinically and organizationally hazardous. We recognized that, despite the absence of public harm, the risk to public safety is increased by forensic patients who are AWOL; some of these patients had committed an index offense involving serious violence, and were also judged to be at elevated risk for future violence [13]. This highlights the urgency of developing an empirically-guided intervention strategy to reduce absconding, and studying its effects on the prevalence and characteristics of absconding events, as well as the risk of harm posed by the patients involved in them.

Results from the current investigation demonstrated that the implementation of a new policy guided by structured professional judgment principles and team-based decision making had a significant impact on the rate of absconding over time. Specifically, both a patient- and event-based index of absconding demonstrated a one-third reduction in the rate of absconding when the 18 months prior to the policy implementation were compared to the 18 months following implementation. The new policy required clinical teams to systematically consider risk factors from the HCR-20 in the domains of prior rule/supervision violations, substance misuse, insight, psychiatric stability and treatment compliance when making decisions regarding patient leave and changes in privilege level. This selection of risk variables was informed by prior investigations [8,13,36-38] showing that patients who abscond were more likely to have had prior absconding attempts and a diagnosed substance use disorder, and that their AWOLs frequently involved the use of substances. We extended this to include recently active and dynamic indicators of risk (e.g., recent problems with substance use, insight and treatment compliance) to reflect the demonstrated ability of these variables to predict adverse outcomes in the short-term. Further, a significant proportion (35%) of absconding patients were characterized as having gone AWOL as a direct result of active or changing psychiatric symptoms, underscoring the role of psychiatric stability and recent treatment compliance in the assessment of risk for absconding.

In addition to risk factors, teams were also required to explicitly consider the purpose of the leave being requested, and describe its connection to the rehabilitative goals of the patient. They were asked to define the risks and benefits of the leave, including any active victim-related issues as well as the patient’s attitude towards receiving versus not receiving the leave. These additional decision-making steps appeared to effectively turn the leave granting process into a more deliberate, systematic and transparent one. Furthermore, given the existing literature suggesting much variability in the procedures and process surrounding leave decision-making [27,28], it is possible that our new policy helped to regulate and systematize this process, ultimately resulting in more consistent and risk-informed decisions. As the current investigation represents the first 18 months following implementation of this new policy, we anticipate continued declines in the rate of absconding as clinical teams become increasingly comfortable and adept at the process. At the same time, research attesting to the difficulty of reducing rates of absconding over time [35], even alongside increasing emphasis on risk assessment and management practices [39], is important to recognize.

Results also suggest that the new policy helped reduce noncompliance with medication and existing privilege levels among patients, at least as measured in the month prior to an incident of absconding. The observed decreases in changes made to existing medication and privilege levels implies a reduction in the contribution of active psychiatric symptoms and noncompliance in the absconding incidents that continued to occur as the policy reached full implementation. This finding also suggests an increase in consistency among clinicians making medication- and leave-related decisions as the policy came into effect. In contrast, the observed increase in threatening/violent behaviors during and following the policy implementation was unexpected, but merges with our finding of ongoing, high levels of frustration and anger as privileges were initially restricted, and then monitored more formally under the new policy. Further, the finding that patients expressed ideation to abscond prior to one-quarter of all incidents, and that this remained unchanged across the study window, suggests that ongoing improvements in communication between patients and clinical teams must occur.

We found a differential impact of the policy on certain types of unauthorized leaves, whereby the frequency of absconding from unaccompanied hospital grounds and community passes was most reduced. Previous studies have noted that the majority of absconsions occur after a patient has already been granted permission to leave the ward or hospital grounds [40-42]. We found this to be true in our setting as well, and so it is relevant that the new policy was able to achieve significant effect in reducing this frequent and common type of absconding behavior. Unfortunately, however, this effect was offset by a small but significant increase in the rate of absconding from within a secure unit, or from a staff accompanied pass. This may have been foreseeable to some degree, to the extent that patients with comparatively lower privilege levels (i.e., those with no leave access or those requiring staff accompaniment) grew increasingly frustrated during the initial policy implementation when leave requests were held and then reviewed with increased scrutiny. Nevertheless, the increase in absconsions from secure units and accompanied privileges is a concern, given that these patients have been judged as being of higher risk to others and necessitating higher levels of supervision. These results underscore the importance of continuing review on thresholds for granting leave and matching patient risk level with adequate security arrangements. They may also indicate a need for a modified decision-making process and/or additional considerations for higher risk patients.

The characteristics of patients who absconded across the study window did not appear to change appreciably as the policy was implemented; however, patients with multiple absconsions were observed to populate more than one study window, making it difficult to ascertain differences between groups of patients who absconded prior to, versus following, policy implementation. When compared to patients with no absconding events, however, those who absconded at any time period were found to have spent significantly more time under the auspices of the forensic mental health system. Consistent with prior research, these patients were more likely to have a history of problematic substance use and problematic personality features, as well as a history of attempted absconding [8,36-38]. Patients who absconded were also estimated to be at higher risk for future violence as per the HCR-20.

We continued to find a widespread sense of frustration and boredom underlying patients’ absconding behavior. Over 50% of absconding events in each time window studied could be characterized by a primary motive involving frustration and/or boredom, often related to lengthy periods of detention without being allowed greater freedoms. As mentioned above, it is possible that the new policy may have initially amplified these sentiments, particularly among higher risk patients with already limited privilege levels. These results underscore how sentiments of frustration and boredom within a forensic setting are particularly challenging clinical issues to address effectively, but at the same time may hold the greatest promise in terms of reducing rates of absconding. In contrast to the existing literature [e.g., 14], the next most common motivating influence we found in this sample pertained to absconders’ psychiatric symptoms. Implementation of our new policy was seen to have a modest impact in reducing the frequency of symptom-driven absconding, suggesting that the new procedures helped teams identify potential risks associated with allotting increased leave privileges to patients with active symptoms and/or instability in mental status.

One of the major benefits of this study was the creation of a new tool designed to assist clinical teams in making leave decisions, as well as decisions related to changing hospital grounds/community access privileges for patients. As noted, there currently exists no structured decision-making tool designed to assess a patient’s risk for absconding. The development of the current tool in this study reflects a first step in this direction, using an already established violence risk assessment scheme to assess relevant domains of risk for absconding behavior specifically. The effectiveness of this tool in reducing observed rates of absconding following its initial implementation is promising, and supports the validity and utility of the selected HCR-20 variables in assessing risk for absconding. The additional steps involved in completing the tool resulted in decisions that appeared more consistent, transparent, and justifiable, as well as informed by a multidisciplinary team, factors which likely improved their overall quality and ability to distinguish patients who could or could not be afforded leave.

The current study is not without limitations. Our measure of absconding rate was a relatively coarse one, and the information which formed the basis of our analyses was taken from the electronic health record. We did not specifically interview patients about why they absconded, which could have provided more detailed information surrounding the motivational aspect of the behavior. Further, our sample size was modest when divided by the three time windows, which could have impacted power to detect smaller sized effects.

The statement made by Bowers and colleagues [6], that “there are no thoroughly convincing, well designed, rigorously carried-out trials of interventions to reduce absconding” (p. 350), is still true. The current study represents an initial evaluation of a new policy designed to reduce absconding in a forensic setting, and we will continue to evaluate its effects over time. Further studies are required to evaluate similar types of policies and tools in secure settings. Fortunately, the HCR-20 is the most widely used structured professional judgment tool for assessing violence risk, and the current results show it has utility in informing decisions about risk for absconding. Consequently, future investigations may also look to the HCR-20 in formulating leave policies and constructing risk assessment schemes for absconding.

## Conclusion

The current study is the first to demonstrate the effectiveness of a policy informed by structured professional decision-making and empirically derived risk factors for violence in reducing rates of absconding in a forensic setting. In contrast to older studies conducted in high secure settings [8-10,19,20], the current setting is more reflective of many modern forensic rehabilitation facilities, and in our case, one that is closely integrated with the downtown community of a large city. Arguably, attempts to manage and reduce absconding in this type of setting present with unique challenges not present in older forensic hospitals that tended to be far removed from urban city centres. Our analysis of patient-reported motivations continued to reveal a wide-spread sense of boredom and frustration with the forensic system, but also showed ongoing heterogeneity in the primary motives underlying the decision to abscond. In light of this, we would expect to see further reductions in the rate of absconding if our new policy and procedures around granting leave could be refined to include clinical assessments and interventions around these motivational influences.

## Additional file

Additional file 1:
**Leave application form.**
