# Supplementary material for: The impact of structured decision making on absconding by forensic psychiatric patients: results from an A-B design study
Source: BMC Psychiatry. 2015 May 3;15:103. doi: 10.1186/s12888-015-0474-1 (PMC4424885; doi:10.1186/s12888-015-0474-1)
Supplement: Additional file 1: — Leave application form. [file 12888_2015_474_MOESM1_ESM.pdf]

camh
Centre for Addiction and Mental Health

* SAMPLE *

Client/Patient ID Label

LEAVE APPLICATION FORM
(ORB PRIVILEGE REQUEST)

Must be accompanied by an ORB Privilege Authorization.

Client/Patient Name: ______________________ (last name, first name)

Date of Team Discussion: ______________________

Unit/Clinic/Service: ______________________

Health Record #: ______________________

Index Offence (with date):	
Date of last AWOL:	
Pass Request (what are they asking for): *attach ORB Privilege Authorization form with details of request.	☐ New Pass ☐ Reinstatement
**Dynamic Risk Assessment (HCR-20, V2)**	**Score /Context /Comments (see HCR-20 manual for scoring guide)**
H5 - Substance Use Problems (please comment)	
H10 - Prior Supervision Failure (please comment)	
C1 - Lack of Insight (please comment)	
C2 - Negative Attitudes (please comment)	
C5 - Unresponsive to Treatment (please comment)	
R4 - Noncompliance with Remediation Attempts (please comment)	
**Current Clinical Situation**	**Context/Comments**
1. Given the above risk factors, what strategies have been put in place to mitigate the risk?	
2. What is their current level of privilege, and how long have they been successfully using it?	
3. If past AWOL, how does the client/patient view it now?	
4. Are there other recent incidents or concerns that need to be considered (i.e. increased agitation, changes in behaviour)?	
5. Possible negative impacts of not granting the pass request?	

Centre for Addiction and Mental Health

Client/Patient ID Label

ORB PRIVILEGE REQUEST

Client/Patient Name: ______________________ (last name, first name)

Date of Team Discussion: ______________________

Unit/Clinic/Service: ______________________

Health Record #: ______________________

**Purpose of Pass**	**Context/Comments**
1. What is the purpose of the pass request?	
2. How does the person view the request?	
3. Is it part of a clear rehab plan that they are engaged in?	
4. What are the related goals in the IPCC?	
Comments from team discussion:	

Request recommended by:

Attending Physician/Resident:

______________________ (signature) ______________________ (print name and credentials) Date: ______________________ (dd/mm/yyyy)

Assigned Nurse:

______________________ (signature) ______________________ (print name and credentials) Date: ______________________ (dd/mm/yyyy)

Manager:

______________________ (signature) ______________________ (print name and credentials) Date: ______________________ (dd/mm/yyyy)

Please submit to the Office of the Person in Charge (OPIC) with a completed and signed ORB Privilege Authorization form. The team will receive OPIC approval or further questions within one week.

Comments and feedback from OPIC	
